# Comparison of idiopathic and secondary uveitis patients seen in a dual pediatric rheumatology-ophthalmology clinic

**DOI:** 10.1186/1546-0096-10-S1-A80

**Published:** 2012-07-13

**Authors:** Victoria TE Rizk, Mara L Becker, Mark F Hoeltzel, Scott E Olitsky, Andrew Lasky

**Affiliations:** 1 Children's Mercy Hospital, Kansas City, MO, USA

## Purpose

Uveitis is an intraocular inflammatory disease that accounts for 10-15% of all cases of total blindness in the U.S. Although uveitis is more prevalent in adults, children comprise approximately 5% of the uveitis population. The most common etiologies of childhood uveitis are idiopathic, infectious, and rheumatologic. Idiopathic uveitis is the most common cause of pediatric uveitis, while uveitis associated with Juvenile Idiopathic Arthritis (JIA) is the most common secondary cause. In 2005, the physicians of Children’s Mercy’s Hospital created a dual Pediatric Rheumatology and Ophthalmology uveitis clinic for patients with ocular inflammation requiring systemic immunosuppressant therapy. An ophthalmologist and rheumatologist jointly evaluate each patient, and formulate a comprehensive plan with the patient and family. To our knowledge, this is the only pediatric uveitis clinic in the U.S. staffed by the two subspecialties simultaneously. The aim of this study was to determine if the characteristics of ocular involvement differ between severe Idiopathic uveitis (IU) and Secondary uveitis (SU).

## Methods

A retrospective study of patients identified through a scheduling database that were evaluated in the uveitis clinic from February 2005 to January 2010 was conducted. The patients were subcategorized into two groups: IU and SU based on the association or lack thereof with an underlying autoimmune disease. Clinical characteristics recorded included the involved site of inflammation, laterality, visual acuity, complications defined as ocular structural damage and pressure changes, concomitant number and type of systemic medications needed to control inflammation, and surgical interventions. Frequencies were compared between groups using Chi-square analysis.

## Results

Of the 41 patients seen over 456 encounters, 29 had IU (71%) and 12 had SU (29%). Of the 12 SU patients, 10 (83.3%) had JIA, 6 oligoarticular and 4 polyarticular, 1 (8.3%) had Sarcoidosis, and 1 (8.3%) had Hashimoto’s. Overall, the maximum number of systemic immunosuppressants used concomitantly was 4. Throughout the course of treatment, patients received the following medications: methotrexate (95%), TNF-a inhibitors (34%), cyclosporine (22%), and mycophenolate mofitil (15%). Surgical procedures over the course of follow-up, most commonly vitrectomy and cataract removal, did not differ significantly between groups.

**Table 1:** 

At presentation:	Idiopathic-29	Secondary-12	P value
Site of involvement			
-Chronic anterior	18 (62%)	9 (75%)	
-Pars planitis	7 (24%)	3 (25%)	
- Panuveitis	2 (7%)	0 (0%)	
-Acute anterior	1 (3%)	0 (0%)	
-Chronic posterior	1 (3%)	0 (0%)	0.76

Laterality			
- Bilateral	19 (66%)	10 (83%)	
- Unilateral	10 (34%)	2 (17%)	0.25

Visual acuity >20/50 in worst eye	10 (36%)	2 (17%)	0.23

Visual acuity >20/200 in worst eye	7 (25%)	1 (8%)	0.23

Complications			
-Synechiae	6 (21%)	2 (17%)	0.77
-Band keratopathy	4 (14%)	1 (8%)	0.63
-Ocular hypertension	5 (18%)	4 (33%)	0.28
-Cataracts	3 (10%)	0 (0%)	0.25

## Conclusion

No statistically significant differences were found in the clinical characteristics and outcomes in patients with IU and SU requiring systemic immunosuppressive therapy. This could be due to a limited sample size, however, these subsets of severe patients may be more phenotypically similar than previously recognized. This may allow for the combination of both subtypes in future studies of childhood uveitis.

## Disclosure

Victoria T. E. Rizk: None; Mara L. Becker: None; Mark F. Hoeltzel: None; Scott E. Olitsky: None; Andrew Lasky: None.

